# Genetic decline and recovery of a demographically rebuilt fishery species

**DOI:** 10.1111/mec.16697

**Published:** 2022-10-11

**Authors:** Jennifer A. Hoey, Kenneth W. Able, Malin L. Pinsky

**Affiliations:** ^1^ Ecology, Evolution, & Natural Resources Rutgers University New Brunswick New Jersey USA; ^2^ Institute for Biodiversity Science and Sustainability California Academy of Sciences San Francisco California USA; ^3^ Marine Field Station, Department of Marine and Coastal Sciences, Rutgers University Tuckerton New Jersey USA

**Keywords:** demographic history, effective population size, genetic variation, recovering fishery, summer flounder

## Abstract

The demographic history of a population is important for conservation and evolution, but this history is unknown for many populations. Methods that use genomic data have been developed to infer demography, but they can be challenging to implement and interpret, particularly for large populations. Thus, understanding if and when genetic estimates of demography correspond to true population history is important for assessing the performance of these genetic methods. Here, we used double‐digest restriction‐site associated DNA (ddRAD) sequencing data from archived collections of larval summer flounder (*Paralichthys dentatus*, *n* = 279) from three cohorts (1994–1995, 1997–1998 and 2008–2009) along the U.S. East coast to examine how contemporary effective population size and genetic diversity responded to changes in abundance in a natural population. Despite little to no detectable change in genetic diversity, coalescent‐based demographic modelling from site frequency spectra revealed that summer flounder effective population size declined dramatically in the early 1980s. The timing and direction of change corresponded well with the observed decline in spawning stock census abundance in the late 1980s from independent fish surveys. Census abundance subsequently recovered and achieved the prebottleneck size. Effective population size also grew following the bottleneck. Our results for summer flounder demonstrate that genetic sampling and site frequency spectra can be useful for detecting population dynamics, even in species with large effective sizes.

## 1 INTRODUCTION

Effective population size (*N*_e_) quantifies genetic drift in a population, making it one of the most important parameters in conservation and evolutionary biology (Charlesworth, 2009). As *N*_e_ declines, the rate of genetic drift increases, decreasing the amount of standing genetic variation in a population and reducing the effectiveness of selection, all of which can limit a population's evolutionary potential (Kelly et al., 2013; Lai et al., 2019; Messer & Petrov, 2013). Especially in today's changing world, *N*_e_ is an important predictor of the repertoire of responses available within a population to overcome novel environmental challenges. As a result, determining whether and when *N*_e_ changes over time and how changes in *N*_e_ correspond to the demographic history of the population remain key priorities in the fields of conservation and evolutionary biology (Díez‐del‐Molino et al., 2018).

Since it is challenging to collect enough demographic information to estimate *N*_e_ directly, a rich area of research has focused on the development and evaluation of indirect genetic estimators of *N*_e_ (Luikart et al., 2010). Currently, the most common methods to estimate *N*_e_ include the linkage disequilibrium and temporal methods (Hill, 1981; Jorde & Ryman, 1995; Krimbas & Tsakas, 1971; Waples et al., 2014). Linkage disequilibrium methods work relatively well for populations with small effective population sizes (*N*_e_ < 1000) if enough individuals are sampled, but once effective size becomes large (>1000), robust estimates of *N*_e_ are challenging to obtain and difficult to interpret (Marandel et al., 2019). With large populations, the genetic diversity metrics (i.e., inbreeding, heterozygosity, linkage, and allelic diversity) that are often used to infer population size differ little across a large range of population sizes, resulting in lower precision for larger *N*_e_ estimates (Palstra & Ruzzante, 2008). This has made the estimation of *N*_e_ and the detection of changes in *N*_e_ particularly difficult for large marine populations, which often have a million or more individuals (Hare et al., 2011). To improve *N*_e_ estimates when employing these methods, suggestions have been made to use exceptionally large numbers of individuals (e.g., 1% of all individuals in a population) and many loci (Marandel et al., 2019; Waples et al., 2018; Waples & Do, 2010). Methods that employ the site frequency spectrum (SFS) of a single population—or the joint (or multisample) SFS for two (or more) populations— have shown promise for detecting changes in *N*_e_ over time (Adams & Hudson, 2004; Excoffier et al., 2013; Gutenkunst et al., 2009; Nunziata et al., 2017; Nunziata & Weisrock, 2018; Patton et al., 2019). Power to detect changes can be particularly high if archived specimens are available to sample a population through time (Nunziata et al., 2017; Nunziata & Weisrock, 2018; Ramakrishnan et al., 2005).

Methods that utilize the SFS have become increasingly popular due to the creation of tractable computational frameworks for estimating the SFS for arbitrary demographic histories (Excoffier et al., 2013; Gutenkunst et al., 2009) and the ease of generating sequencing data for many individuals at thousands of loci. The SFS is a count summary of the number of derived or minor alleles in each of the sampled populations and is particularly useful when all loci are biallelic. The distribution of alleles in the SFS, which is related to the rate at which lineages merge, or coalesce, is indicative of the evolutionary history of the population(s) under consideration, including changes in population size and migration events. In general, an excess of rare alleles in the SFS indicates rapid population expansion (Keinan & Clark, 2012), while a deficit of rare alleles may indicate a recent population bottleneck because rare variants are lost disproportionately quickly due to genetic drift (Maruyama & Fuerst, 1985). During a population bottleneck, faster than expected rates of coalescence will result in fewer rare variants. On the other hand, growing population sizes and slower coalescence rates produce larger numbers of rare variants (Gattepaille et al., 2013). In theory, multiple demographic scenarios can result in the same SFS (Myers et al., 2008), so distinguishing among similar scenarios can be challenging. However, modelling of biologically realistic demographic scenarios suggests that underlying demography can often be identified from the SFS, especially when enough individuals have been sampled (Bhaskar & Song, 2014). SFS‐based methods have been successfully applied to a number of real data sets to understand past changes in population size (Harris et al., 2016; Keinan & Clark, 2012; McCoy et al., 2014; Nunziata et al., 2017; Sovic et al., 2019), and these methods may be particularly good for understanding changes on contemporary timescales up to 30 generations ago (Nunziata et al., 2017; Nunziata & Weisrock, 2018; Patton et al., 2019).

Effective population size can be estimated over long or short time scales, with each having its own utility for practical management and conservation goals (Hare et al., 2011). Estimates of effective population size in deep time (hundreds to thousands of years) are useful for placing modern populations within a historical context (Harris et al., 2016; Huff et al., 2010; Roman & Palumbi, 2003), but contemporary effective population size estimates are more relevant for predicting persistence and for guiding management decisions (Luikart et al., 2010).

Populations with well‐known demography are critical for assessing the robustness of contemporary effective population size estimates because they provide a direct comparison between population estimates using genetic data and those using more traditional sampling techniques (McCoy et al., 2014; Nunziata et al., 2017). Harvested and managed fishes represent some of the most well studied natural populations, and with a wealth of data over time, provide key opportunities for understanding how historical demographic processes influence genetic variation and effective population size on a contemporary time scale. Theory suggests that intensive harvest can induce a genetic bottleneck, and fishing is expected to reduce genetic diversity (Hauser et al., 2002; Hutchinson et al., 2003; Pinsky & Palumbi, 2014; Therkildsen et al., 2019). Yet, how the timing and magnitude of genetic declines and recovery correspond to demographic bottlenecks and recovery remains largely unexplored in harvested populations (Kuparinen et al., 2016). The large population sizes of many fishery species make estimation of *N*_e_ challenging using linkage disequilibrium or genetic diversity methods, but such species provide an opportunity to test if SFS‐based methods might be particularly well‐suited for large populations. In addition, while the genetic theory for demographic inference is relatively clear, natural populations rarely match all assumptions of theoretical methods. Therefore, opportunities that allow for comparing known population demography against estimates of contemporary effective population size over time provide a promising avenue for testing the utility of genetic monitoring in wild populations (Schwartz et al., 2007).

Of the 450+ managed U.S. marine fish stocks and stock complexes, 45 were rebuilt to their targeted abundance levels between 2000 and 2018 and another 43 still required rebuilding at the end of 2018 (NOAA Fisheries, 2019). One such recovered stock was summer flounder (*Paralichthys dentatus*), an ecologically and economically important species in the Mid‐Atlantic region of the U.S. East coast. Terceiro (2001) suggested that summer flounder biomass was low in the 1960s before doubling in size between 1967–1974. Peak commercial landings then occurred in 1979, followed shortly thereafter by an estimated 77% decline in spawning stock biomass from approximately 53 million pounds in 1982 to 12 million pounds in 1989 (Terceiro, 2001). Since then, a strong focus on management for rebuilding helped spawning stock biomass increase again to a high of 110 million pounds in 2003 (an estimated 800% increase from 1989) before tapering off and declining slightly in the present (Terceiro, 2016). Starting in 1989 and 1985, the Rutgers University Marine Field Station and the NOAA Beaufort Laboratory, respectively, have collected larval summer flounder on a weekly basis as the larvae ingress into estuaries that serve as nurseries. These collections represent an unprecedented opportunity to uncover how genetic diversity and effective population size changed in response to dramatic changes in census population size in an exploited but demographically recovered marine population.

Here, we used double‐digest restriction‐site associated DNA (ddRAD) sequencing data from archived collections of larval summer flounder (*n* = 279) from three serially sampled larval cohorts (1994–1995, 1997–1998 and 2008–2009) along the U.S. East coast to empirically estimate effective population size and genetic diversity just after a population decline and during a recovery period following the reduction of intense fishing pressure. Understanding how *N*_e_ and genetic diversity respond to a population bottleneck and subsequent recovery can allow insight into whether summer flounder may be genetically limited in their response to future perturbations. Using summer flounder as a case study, we ask: (1) How does a severe demographic decline and recovery empirically affect genetic diversity and contemporary effective population size over time in a harvested population? (2) To what extent do contemporary genetic estimates of demographic history match known changes in census population sizes in a natural population?

## 2 MATERIALS AND METHODS

### 2.1 Abundance estimates at peak spawning from fisheries data

Standardized fisheries trawl surveys have been conducted since 1963 in the waters off the northeastern U.S. (Azarovitz, 1981). These data are incorporated into stock assessment models to calculate spawning stock biomass, abundance at age, the proportion of mature fish in each age class, mortality due to fishing and natural causes, and other demographic parameters. Using data from the 2016 summer flounder stock assessment (Terceiro, 2016), we calculated total abundance of breeding adults at peak spawning (*N*_*ps,t*_) for each year from 1982–2015 using $N_{\mathrm{ps},t}=\sum_{a=0}^{A}N_{a,t}e^{-pZ_{a,t}}$, where *p* = 10/12 was the fraction of the year that had passed when peak spawning occurred (around November 1 for summer flounder), *Z*_*a,t*_ was total mortality (natural mortality + fishing mortality) for age class *a* in year *t*, *N*_*a,t*_ was the number of sexually mature breeding adults in a given age class at the beginning of the year, and *A* was the oldest age class.

### 2.2 Larval collections

**FIGURE 1:**
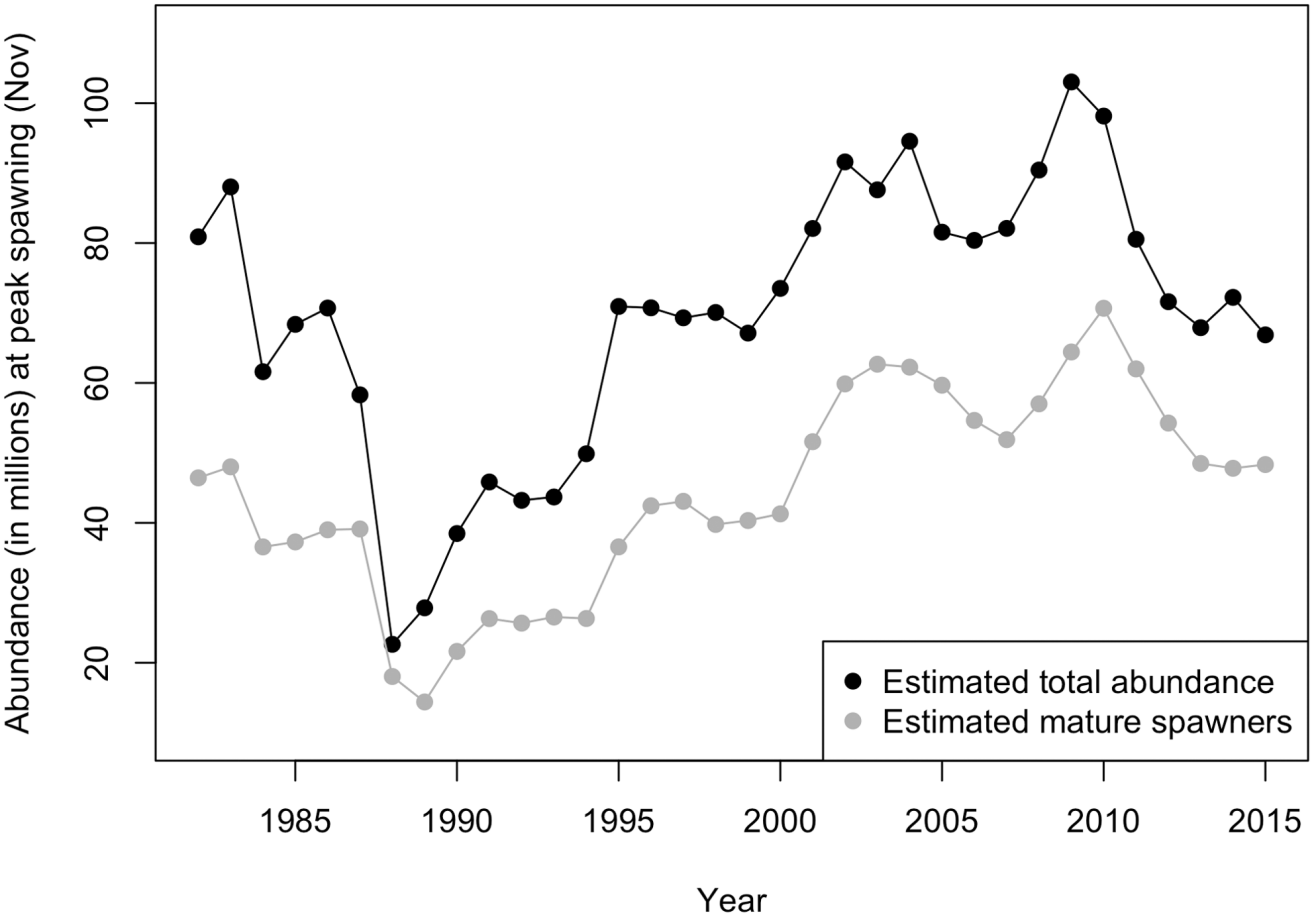
Abundance estimates of total population size and number of mature spawners at peak spawning time from the summer flounder stock assessment (Terceiro, 2016).

Larval summer flounder have been collected at the Rutgers University Marine Field Station (RUMFS, Little Egg Inlet, New Jersey) on a weekly basis since 1989, with fish assemblages from this sampling site being representative of much of the New Jersey (NJ) coastline (Able et al., 2011, 2017). Summer flounder larvae ingress into shallow bays and estuaries, with the peak occurring between October–December and continuing through April in New Jersey (Able et al., 1990; Keefe & Able, 1993). Based on this timing, we defined a larval collection cohort year as beginning in the fall (October–December) and extending into the winter (January–March) months. We sampled three larval cohort years to examine how contemporary *N*_e_ had changed over time: Fall 1994–Winter 1995 (1994 cohort), Fall 1997–Winter 1998 (1997 cohort) and Fall 2008–Winter 2009 (2008 cohort). Each sampled larval cohort represented a snapshot of the adult summer flounder that contributed alleles to the next generation. These years were selected as time periods when summer flounder population size was low, growing, and high, respectively (Figure 1).

Larvae sampled from RUMFS were pooled with additional larvae captured at the NOAA Beaufort (North Carolina) Laboratory from the corresponding larval cohort year (Table 1). These North Carolina (NC) larvae were initially sampled for another project, but because the summer flounder population is effectively panmictic due to high dispersal (Hoey & Pinsky, 2018) and larvae disperse across Cape Hatteras, NC frequently (Hoey, Fodrie, et al., 2020), we concluded that including NC larvae to increase our sample size was appropriate for investigations of *N*_e_ and genetic diversity.

**TABLE 1:** Genetic diversity statistics calculated for summer flounder cohorts. Included are the number of fish from each cohort (Total) and from each ingress location within a cohort (NJ, New Jersey or NC, North Carolina), the average observed heterozygosity per locus (Het_obs_), expected heterozygosity (Het_exp_), Wright's inbreeding coefficient (*F*_IS_) and nucleotide diversity (π) with bootstrapped 95% confidence intervals

Cohort and capture location	No. of fish	Het_obs_	Het_exp_	*F*_IS_	π	π 95% CI
1994–1995: Total	26	0.0654	0.0694	0.0581	0.00457	0.00429–0.00457
Little Egg Inlet, NJ	26					
Beaufort, NC	0					
1997–1998: Total	103	0.0651	0.0688	0.0543	0.00469	0.00455–0.00483
Little Egg Inlet, NJ	85					
Beaufort, NC	18					
2008–2009: Total	150	0.0669	0.0698	0.0415	0.00378	0.00367–0.00390
Little Egg Inlet, NJ	138					
Beaufort, NC	12					

### 2.3 DNA extraction, library preparation & sequencing

For all larval summer flounder samples, the posterior portion of the body was used for DNA extraction using DNeasy 96 Blood & Tissue Kits (Qiagen) and the manufacturer's recommended protocols. Individuals were randomly distributed among 96‐well plates for extractions. DNA extracts were visualized on 2% agarose gels to assess quality and were subsequently quantified using PicoGreen (Thermo Fisher Scientific) and a SpectraMax M3 Microplate Reader (Molecular Devices).

Summer flounder ddRAD libraries were prepared according to a protocol adapted from Peterson et al. (2012) and described in detail in Hoey and Pinsky (2018). Briefly, successful extracts were digested in 50 μl reactions using PstI and EcoRI restriction enzymes for 4 h at 37°C. Digested samples were cleaned with AMPure beads (Beckman Coulter) to remove small DNA fragments less than 100 base pairs (bp) in size and any remaining proteins, including restriction enzymes. Cleaned digestions were then ligated to P1 and P2 adapters. The P1 adapter contained individual barcodes. Ligated samples were pooled and cleaned before being size selected to a mean size of 273 ± 27 bp using a Blue Pippin or Pippin Prep (Sage Science), and then amplified using PCR read 1 and read 2 primers specifically designed to only amplify DNA with both P1 and P2 adapters. Read 2 PCR primers contained one of 12 Illumina indices so that pools could be distinguished from one another. PCR products were cleaned and Qubit fluorometric quantitation (Thermo Fisher Scientific) was used to quantify the final concentration of each pool.

Library preparation for larvae sampled from the 1994 and 1997 cohorts (historical) was performed in laboratory facilities in separate buildings (Marine and Coastal Sciences Building and Waksman Institute, Rutgers University) from those in which larvae from the 2008 cohort (modern) were processed (Environmental and Natural Resources Building, Rutgers University). Care was taken to not bring equipment, reagents or clothing between the laboratories in order to limit contamination of our historical samples by modern fish DNA. For samples collected in 1998 and prior (historical), we randomly introduced at least one blank control for every 24 individuals during the extraction and digestion steps and then carried these blank controls through to sequencing. In addition, unique P1 adapters were utilized for historical samples during the ligation step. These precautions provided an additional level of confidence that cross‐contamination between historical and modern samples did not occur.

Laboratory work was completed between 2015 and 2018. Pools of 24–48 individuals comprised three DNA libraries that were sent to the Princeton Genomics Core Facility (Princeton, NJ) for 140 to 150 bp single‐end sequencing on two‐lane runs using the Illumina HiSeq 2500 platform. In all, 331 larval summer flounder were sequenced for this study.

### 2.4 Bioinformatics and genotyping

To distinguish between pooled libraries, sequenced reads were demultiplexed by Illumina index using a Python script adapted from FASTX Barcode Splitter (Gordon, 2011). Sequenced reads were further demultiplexed by barcode and cleaned using process_radtags in STACKS version 1.29 (Catchen et al., 2013). Sequences were then run through dDocent version 2.8.12 (Puritz et al., 2014), an analysis pipeline for ddRADseq data that is described next. First, all reads were cropped to 140 bp (the lowest common read length among sequencing runs) and trimmed for quality using Trim Galore! (Krueger, 2015). BWA (Li, 2013) was used to map individual larval quality‐trimmed reads to a de novo single‐end ddRADseq reference assembly built from a sequencing run containing 351 larval individuals with 150 bp read lengths (299 of which were used in this study, plus 52 sequenced for a separate study that were captured between 1990–1993 and 2010–2012 from NJ and NC). Reference assembly was performed with Rainbow (Chong et al., 2012) using alleles with a minimum within‐individual coverage level of 4 and a minimum occurrence in 15 individuals. Reference sequences with >90% similarity were clustered together using CD‐HIT (Fu et al., 2012; Li & Godzik, 2006). Following read mapping, single nucleotide polymorphisms (SNPs) were identified across all 331 larval individuals from the three cohorts of interest using FreeBayes (Garrison & Marth, 2012).

We retained variant SNPs that were successfully genotyped in at least 50% of individuals with a minimum quality score of 30. We did not employ a minor allele frequency nor a minor allele count filter. Individuals with >50% missing data were discarded (~14% of all individuals). Data were then restricted to variants occurring in 95% of remaining individuals with a minimum mean depth of 20. Further filtering was conducted using the default settings of the dDocent_filters script distributed with dDocent. This script filtered variants based on criteria related to site depth, quality versus depth, mapping quality, strand representation, and allelic balance at heterozygous individuals. Indels were removed, and only the first SNP at each contig was retained in order to help ensure an unlinked data set. These filtering steps resulted in 3905 loci across 284 larvae. To further reduce potential contamination that may have occurred during larval sampling, storage, or DNA library preparation, we calculated the proportion of heterozygous loci within individuals and removed five fish whose individual heterozygosity was three standard deviations above the mean (Petrou et al., 2019). We then identified loci not in Hardy–Weinberg proportions (HWP; *p* < .001) using the pegas version 0.13 package (Paradis, 2010) in R. These additional filters resulted in 3749 loci across 279 larvae for downstream analyses, unless otherwise noted.

### 2.5 Genetic diversity, single‐sample *N*_e_, and selection

Nucleotide diversity (π) across 140 bp windows was calculated using vcftools version 0.1.17 (Danecek et al., 2011) and all available SNPs on a contig for each larval cohort. For within‐cohort estimates of π, 95% confidence intervals were calculated by bootstrapping 1000 times across individuals using the boot version 1.3–24 (Canty & Ripley, 2019) package in R (R Core Team, 2017). Observed and expected heterozygosity per locus and *F*_IS_ were calculated using the *basic.* *stats* function in the hierfstat version 0.04–22 (Goudet, 2005) package in R.

Single‐sample estimates of *N*_e_ were generated for each sampled larval cohort using the linkage disequilibrium method (Waples & Do, 2010) with random mating implemented in NeEstimator version 2.1 (Do et al., 2014). All other options were set to the default. We report point estimates resulting from the removal of singleton alleles and confidence intervals from jackknifing across individuals (Jones et al., 2016).

SNP genotypes were screened for temporal outliers among the three larval cohort years using BayeScan version 2.1 (Foll & Gaggiotti, 2008). BayeScan uses the difference in allele frequencies between samples across space or time to estimate the posterior probability of loci being under selection.

### 2.6 Demographic modelling

**FIGURE 2:**
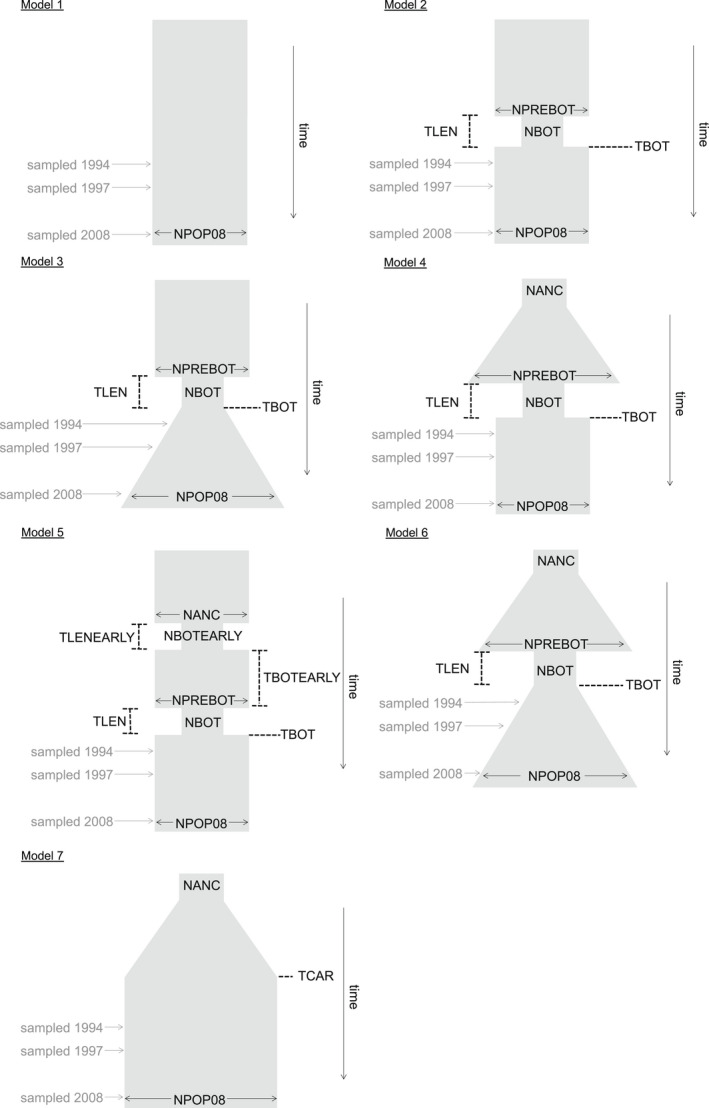
Demographic models with serial sampling for summer flounder: Model 1 constant population size, Model 2 a bottleneck and then an instantaneous change in population size, Model 3 a bottleneck and then exponential growth or decline (depicted here as growth), Model 4 exponential change in population size (depicted here as growth) before a bottleneck followed by an instantaneous change in population size, Model 5 two bottlenecks with instantaneous changes in population size, Model 6 exponential change in population size before and after the bottleneck (depicted here as growth), and Model 7 exponential change in ancestral population size prior to reaching carrying capacity. For the demographic scenarios with instantaneous change in population size, pre‐ and post‐bottleneck sizes could be greater or less than population size during the bottleneck.

We fit demographic models of recent population size changes using a simulation‐based approach and the SFS in fastsimcoal2 version 2.6 (Excoffier et al., 2013, 2021). In addition to the filtering steps mentioned above, we removed all loci with missing data, resulting in 1068 loci across 279 summer flounder individuals. We then summarized these loci across our three larval cohorts as the observed minor allele (folded) multiSFS in Arlequin version 3.5.2.2 (Excoffier & Lischer, 2010). Using fastsimcoal2, we fit parameters for seven demographic models with serial sampling to our observed SFS and estimated the likelihood of our data under each model. Monomorphic sites and mutation rate were ignored during parameter estimation by using the ‐‐removeZeroSFS option. Our seven simple models were chosen to represent the range of likely scenarios that underlie the evolutionary history of summer flounder (Figure 2), including Model 1 a constant population size through time, Model 2 a bottleneck and then an instantaneous change in population size, Model 3 a bottleneck and then an exponential change in population size, Model 4 exponential change in population size followed by a bottleneck and then an instantaneous change in population size, Model 5 two bottlenecks with instantaneous changes in population size, Model 6 exponential change in population size before and after the bottleneck, and Model 7 exponential change in ancestral population size prior to reaching carrying capacity.

Parameters estimated from the models included modern *N*_e_ at the time of sampling in 2008 (NPOP08), *N*_e_ during the bottleneck (NBOT), *N*_e_ just prior to the bottleneck (NPREBOT), the duration and ending times of the bottleneck (TLEN and TBOT, respectively), and the ending time of the ancestral change in population size (TCAR; Figure 2). For Models 4, 6, and 7, we also estimated *N*_e_ (NANC) after the Last Glacial Maximum (Clark et al., 2009). Parameters for the two‐bottleneck model (Model 5) were the same as for the single bottleneck models but were differentiated between the first and second bottlenecks.

We determined female generation length by calculating the average age of females weighted by the number of eggs produced in each age class. Male generation length was calculated assuming that each age class contributed equally to reproduction. Calculations were based off of estimated abundance and the proportion of mature fish in each age class from Terceiro (2016), age‐length relationships from Penttila et al. (1989) and length‐fecundity curves from Morse (1981). Average generation length of females and males from 1982–2008 was calculated to be 2.01 years (Figure S1).

Initial values for the maximum‐likelihood search procedure for population size (NANC, NPREBOT, NBOT, and NPOP08) were log‐uniformly distributed from 100 to 100,000 haploid units; for bottleneck duration (TLEN) were uniformly distributed from 1 to 5 generations; for the end of the bottleneck (TBOT) were uniformly distributed from 1 to 12 generations; and for the end of the ancestral change in population size (TCAR) was log‐uniformly distributed from 1 to 5000 generations. While the lower limit on initial values served as a bound on the search space, the upper limit did not bound the search space. A total of 100,000 simulations were performed to estimate the SFS with a maximum of 40 loops (ECM cycles) for each demographic scenario. For each model, 50 replicate runs with different initial values were performed as single threaded processes on the Amarel Linux computing cluster (Rutgers University), and the overall maximum‐likelihood (ML) was retained. The relative likelihood was compared across models and the best fitting demographic model was selected using Akaike's Information Criterion (Akaike, 1974) after converting the log_10_‐likelihoods reported by fastsimcoal2 to ln‐likelihoods.

We also performed two sensitivity analyses to understand how model specifications impacted our demographic results. First, we expanded the range for TBOT to 1–30 generations and TLEN to 1–15 to test the sensitivity of our results to the initial value ranges. Based on the ML from 50 replicate runs, our parameter estimates did not differ and we did not pursue this sensitivity test further. Following recommendations from fastsimcoal2 to fix one parameter when ignoring monomorphic sites, we also performed a second sensitivity analysis by fixing TLEN at three generations.

Confidence intervals for parameters in the best‐supported model were obtained through non‐parametric bootstrapping. Loci from the observed data set of 1068 loci across 279 larvae were resampled to generate 100 bootstrapped SFS using Arlequin (Excoffier & Lischer, 2010). For each bootstrapped SFS, 30 replicate runs were performed to identify the ML parameter set. The ML parameter estimates for the best‐fit model on the observed data set were used as the starting values for each run in order to efficiently estimate confidence intervals (‐‐initValues). Monomorphic sites were also ignored when estimating parameters for each run (‐‐removeZeroSFS). The set of MLs from the 100 bootstrapped SFS were used to determine 95% confidence intervals for each parameter.

In addition, we performed two sets of simulations to determine the power within our data set for distinguishing among the seven demographic hypotheses. First, we simulated 10 pseudo‐observed SFS for each model with fastsimcoal2 by using the previously obtained ML parameter estimates of each model. We then fit each of the seven models to each of the 70 pseudo‐observed data sets using the initial starting points and run specifications as previously described. Ten replicate runs with different initial values were performed for each model fit to a pseudo‐observed data set. The run with the ML was retained, and AIC was used to determine the best‐fit model for each pseudo‐observed data set. We then compared the best‐fit model to the known generating model to produce a confusion matrix. Second, to help disentangle the effects of temporal sampling from unequal sampling over time, we assessed the power for inferring the correct demographic model when equal numbers of individuals were sampled across cohorts. We simulated 50 pseudo‐observed data sets for the best‐fit model when 1068 loci and 80 diploids in each cohort were sampled. We then fit our seven demographic models to each of the 50 pseudo‐observed data sets. Ten replicate runs with different initial values were performed for each model fit to a pseudo‐observed data set, and the best‐fit model for each pseudo‐observed data set was selected using AIC.

## 3 RESULTS

### 3.1 Genotyping results

The number of quality‐filtered reads per individual was 576,441 ± 626,768 (mean ± SD). Mapping to our reference assembly resulted in an average coverage of 25x per individual. Variant calling across individuals identified 314,570 putative SNPs, and of these, 3905 loci with an average read depth of 61x across 284 larvae passed initial filtering.

### 3.2 Genetic diversity, single‐sample *N*_e_, and selection

Nucleotide diversity (π) across 140 bp windows was lowest in the 2008 larval cohort (π = 0.00378; 95% CI: 0.00367–0.00390) and highest in the 1997 larval cohort (π = 0.00469; 95% CI: 0.00455–0.00483) (Table 1). Observed heterozygosity for each larval cohort ranged from 0.0651–0.0669 and expected heterozygosity ranged from 0.0688–0.0698 (Table 1). Wright's inbreeding coefficient (*F*_IS_) for each cohort varied from 0.0415–0.0581 and declined slightly over time (Table 1), suggesting that inbreeding was highest in the 1994 cohort when summer flounder abundance was reduced. Estimates of *N*_e_ with 95% confidence intervals from NeEstimator were 1168 (365–infinite individuals) for the 1994 larval cohort, infinite (8377–infinite) for the 1997 cohort and 56,672 (5786–infinite) individuals for the 2008 cohort. No temporal outlier SNPs were detected using BayeScan. Therefore, no SNPs were removed prior to demographic modelling.

### 3.3 Demographic modelling

Demographic modelling from serial sampled larval summer flounder strongly supported exponential growth of the ancestral population, followed by a bottleneck, followed by additional rapid exponential growth (Model 6) as the best‐fitting model (Tables S1 & S2). The second‐best model (Model 4) had a ΔAIC of 13 and the third‐best model (Model 7) had a ΔAIC of 22. While the top three models demonstrate clear support for ancient growth up until roughly 10 generations ago, a model containing a subsequent bottleneck followed by an increase in population size was strongly preferred (Tables S1 & S2). Together, these results suggest that historical fishing had a noticeable genetic effect in summer flounder.

The best‐fit demographic model estimated that the ancestral population grew quite slowly (exponential increase of 0.00034 per generation) to 32,209 (95% CI: 9671–57,485) diploid individuals prior to the bottleneck (Table 2 and Figure S2; NPREBOT). The bottleneck lasted two (95% CI: 1–4) generations (Table 2; TLEN) and the end of the bottleneck occurred 12 (95% CI: 8–15) generations prior to 2008 (Table 2; TBOT). When translated into years, the bottleneck occurred from approximately 1980–1984, which aligns well with the low mature spawner census sizes from 1988–1994 (Figure 1). *N*_e_ during the bottleneck was 910 (95% CI: 154–1963) individuals (Table 2 and Figure S2; NBOT). The population then grew rapidly (exponential increase of 0.20 per generation) before reaching a *N*_e_ of 10,212 (95% CI: 5859–37,013) individuals after the bottleneck (Table 2 and Figure S2; NPOP08). The NBOT/NPREBOT ratio was 0.028, suggesting a substantial decline (95% CI: 0.0105–0.0566) and the NPOP08/NBOT ratio was 11.2 (95% CI: 5.27–143), suggesting that the summer flounder population achieved a certain degree of recovery after substantial growth following the bottleneck. The degree to which summer flounder recovered to the prebottleneck effective level can be summarized as NPOP08/NPREBOT. This ratio was 0.317 (95% CI: 0.150–2.95), suggesting some uncertainty in the degree to which summer flounder recovered to the prebottleneck size by 2008.

**TABLE 2:** Maximum‐likelihood (ML) demographic parameter estimates and 95% confidence intervals (CIs) for summer flounder under the best‐fitting demographic model (Model 6: Exponential growth in the ancestral population followed by a bottleneck and then rapid exponential growth in population size). The results from an alternative analysis that fixed TLEN at 3 generations is also presented. Compare to Figure 2 for interpretation of the parameters

Parameter	ML estimate	95% lower CI	95% upper CI	ML fixed TLEN	Unit
NANC	1052	355	1903	581	Diploid individuals
NPREBOT	32,209	9671	57,485	22,972	Diploid individuals
NBOT	910	154	1963	387	Diploid individuals
NPOP08	10,212	5859	37,013	10,171	Diploid individuals
TLEN	2	1	4	3	Length of bottleneck in generations
TBOT	12	8	15	11	Number of generations after bottleneck

**FIGURE 3:**
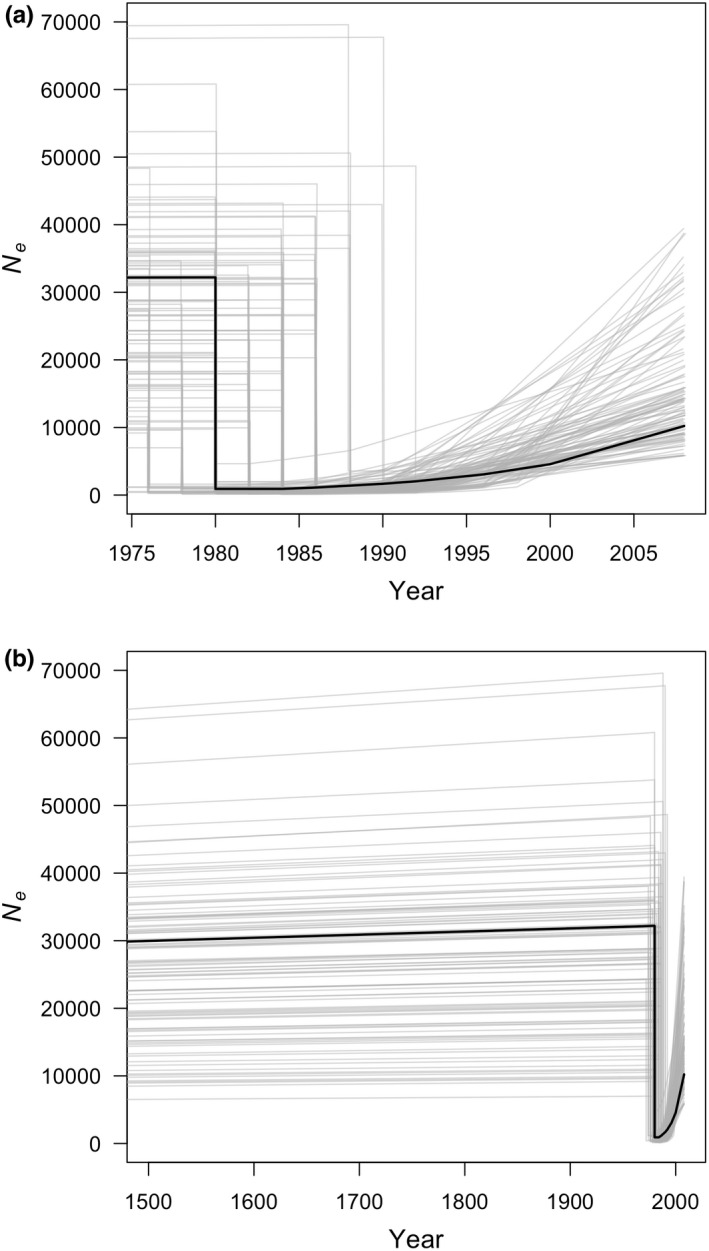
Line plot of estimated effective population size over (a) contemporary and (b) deeper time from the maximum‐likelihood demographic model: Positive exponential growth before and after a bottleneck (model 6; black line). The 100 grey lines in each plot illustrate scenarios used to estimate the 95% confidence intervals for each parameter. Summer flounder generation time was estimated to be 2 years and *N*_e_ estimates have been converted to diploid units.

Overall, the best‐fit model suggests that summer flounder *N*_e_ had been slowly increasing before declining sharply in the early 1980s (Figure 3). The demographic modelling suggested a rapid exponential increase in effective population size after the bottleneck, leading to a noticeable recovery in population size. When TLEN was fixed at three generations (or 6 years) based on summer flounder abundance over time, all parameter estimates were similar to those produced when TLEN was estimated (Table 2).

Simulations revealed high power within the data set for accurately selecting Model 6 (90% probability of selecting Model 6 when it was the true model; Figures S3 and S4). Model 6 was never mis‐identified as Model 4 and occasionally (10% of simulated data sets) mis‐identified as Model 7 (Figure 2). While the ancestral population size changed exponentially in all three of these models, a bottleneck did not occur following this change in Model 7, whereas a bottleneck did occur in both Models 4 and 6. The only difference between Models 4 and 6 was that Model 4 had an instantaneous change after the bottleneck, while Model 6 had an exponential change. There was a high chance of incorrectly selecting Models 6 or 7 (40% probability each) when the true model was Model 4. In contrast, there was a high probability of correctly selecting Model 7 when the true model was Model 7 (80% of simulated Model 7 data sets; Figures S3 and S4). However, there was a low probability of falsely selecting Model 6 when the true model was Model 7 (20%; Figures S3 and S4), suggesting that we can be quite certain of two things: (1) that summer flounder experienced exponential growth of the ancestral population, and (2) that this growth was most likely followed by a bottleneck, followed by additional increase in population size, regardless of whether this increase occurred instantaneously (Model 4) or exponentially (Model 6). Additionally, simulations with equal sample sizes across cohorts suggest high power for accurately selecting Model 6 (88% probability of selecting Model 6 when it was the true model; Figure S5). There was a low probability of incorrectly selecting Models 4 and 7 when the true model was Model 6 (4 and 8%, respectively). This series of simulations provides clear evidence that our temporal sampling scheme resulted in strong inferential power to recover the underlying demographic history.

To further evaluate model fit with temporal sampling, we compared the observed minor allele SFS for each larval cohort with the expected minor allele SFS averaged across 100 SFSs generated using the ML parameters of the three best‐fitting models. The observed SFSs most closely matched with the SFSs expected under Model 6 with exponential growth before and after the bottleneck (Figure S6). However, none of our models could fully explain the relatively high prevalence of alleles with minor allele count 1 in the 1997 larval cohort, but Model 6 came closest to doing so. In addition, none of our models were able to explain both the relatively high prevalence of minor allele counts 2–3 in the 1997 and 2008 larval cohorts, nor the relative rarity of alleles with minor allele counts 4–7 or 7 in the 1997 and 2008 larval cohorts, respectively. In addition, the expected averaged SFSs based on the ML parameters for Model 6 illustrate that the prevalence of rare alleles differed over time, further suggesting that our ability to temporally sample larvae aided in recovering the contemporary demographic history of summer flounder (Figure S7). While differences in the SFS among larval cohorts became less apparent if equal numbers of individuals were sampled in each cohort, small differences were still apparent. In particular, a small but clear pattern of relatively more rare alleles in the earlier cohorts provided additional support that temporal sampling aided in the inference of demographic history (Figure S8).

## 4 DISCUSSION

Effective population size is an important indicator of evolutionary potential, particularly for understanding how species respond to and recover from exploitation. We utilized archived larval summer flounder specimens from periods of low, increasing, and high spawning stock biomass to estimate genetic diversity and to test if SNP data were useful for detecting changes in summer flounder demography. A small decline in genetic diversity was observed between 1997 to 2008, but in general, stable levels of genetic diversity suggested that summer flounder population size has remained relatively large over time. The single‐sample NeEstimator results indicated that *N*_e_ could not be accurately estimated from linkage‐disequilibrium patterns and that the signal could not be distinguished from sampling variance. However, coalescent‐based demographic modelling using the joint site frequency spectrum revealed a substantial decline and subsequent recovery in summer flounder effective population size, consistent with population dynamics recorded by stock assessments of this species (Terceiro, 2001). The timing of the decline in effective population size was also congruent with the timing of the lowest estimates of spawning stock biomass from fisheries data sets, with a difference of only a few generations. Our results in summer flounder suggest that coalescent‐based demographic modelling and SNP‐based SFS data from only a few hundred archived specimens can be a useful strategy for detecting changes in the magnitude and timing of contemporary *N*_e_.

A growing number of studies have employed coalescent‐based demographic modelling and the SFS to estimate *N*_e_ on contemporary time scales (Patton et al., 2019; Sovic et al., 2019), but only a subset have benefited from independent estimates of demography (McCoy et al., 2014; Nunziata et al., 2017). Similar to studies in other organisms that combined coalescent‐based demographic modelling and independent estimates of demography (McCoy et al., 2014; Nunziata et al., 2017), we also detected changes in effective population size that corresponded well with known changes in the census population size of summer flounder. Much like Nunziata et al. (2017), we used serial sampling and SFS‐based demographic modelling to demonstrate that very recent demographic events (~10 generations ago) are detectable. However, our study extends these results to species with a large effective size and more complex historical demography. In particular, the NBOT/NPREBOT ratio indicated a sharp drop in *N*_e_ roughly 10 generations ago following a long period of ancestral growth. In line with the recovery of census abundance, we also found clear evidence for effective population growth and recovery following the bottleneck. Our simulations revealed that distinguishing among Models 4 and 6 could be difficult in some cases, but these models were qualitatively similar and were the top two models during model selection. The only difference between Models 4 and 6 was an instantaneous change in population size following the bottleneck versus an exponential one, respectively. Prior research also suggests that SFS‐based methods are well‐suited to detect recent changes in population size (Nunziata et al., 2017; Nunziata & Weisrock, 2018; Patton et al., 2019), though additional studies will be helpful for assessing the generality of this result.

Whether or not *N*_e_ recovers in line with census abundance is an important question given the influence of *N*_e_ on inbreeding, genetic diversity, evolutionary potential, and other considerations (Kuparinen et al., 2016). An empirical study using a limited number of markers and a theoretical study investigating the consequences of fishing on *N*_e_ have reported that genetic diversity and *N*_e_ can recover following heavy exploitation at the temporal scale of decades (Hutchinson et al., 2003; Kuparinen et al., 2016). Gene flow, population growth, and evolution were proposed as the dominant mechanisms behind these increases. While our study demonstrates that summer flounder effective population size achieved substantial recovery, there remains considerable uncertainty in the exact magnitude. This could be because not enough time has passed for recovery to be fully reflected in the SFS and/or because higher sample sizes are needed for very recent events. In general, parameters for recent demographic events are more challenging to estimate than for ancient events (Adams & Hudson, 2004; Robinson et al., 2014). This is because the timing and intensity of historical events strongly influence the shape of the SFS, particularly the distribution of rare alleles that are important for demographic inference. For example, Gattepaille et al. (2013) found that the SFS tends to remain deficient in rare alleles long after a bottleneck strength of 80%, but with a strength of 95%, the deficit of rare alleles quickly turns into an excess for the very rarest variants, even when the bottleneck is young. These results suggest that signatures of historical demographic events can persist in the SFS, which could obscure signatures of more recent events. In particular, scenarios involving a population bottleneck followed by expansion can be challenging to detect from the SFS (Adams & Hudson, 2004; Nunziata et al., 2017; Robinson et al., 2014), though our temporal sampling design revealed high power to detect such a decline and recovery. Rare alleles can be indicative of population expansion, but they are also quickly lost during a population bottleneck (Maruyama & Fuerst, 1985). The relative rarity of alleles with a minor allele count of 1 in the 2008 cohort might be partially reflective of the slow rate at which rare alleles are regenerated through mutation or the challenge of detecting rare alleles using a genotyping‐by‐sequencing approach (e.g., RADseq). Sampling more individuals or additional cohorts from more recent years could result in more precise estimates of *N*_e_ change after the bottleneck (Keinan & Clark, 2012; Robinson et al., 2014), but theory has demonstrated diminishing returns on the accuracy of SFS‐based inferences as sample size increases for a given number of SNPs (Terhorst & Song, 2015). Rather, increasing the number of SNPs may be more important for improving the precision around estimates of recent demographic change. For example, Nunziata and Weisrock (2018) found that the coalescent‐based method required many SNPs (25,000–50,000) for accurate inference. However, the coalescent method required substantially fewer individuals (on the order of 20) than methods based on linkage disequilibrium that would require about 1% of the census population (Marandel et al., 2019; Nunziata & Weisrock, 2018). In summer flounder, 1% of the census population would be nearly 200,000 samples. Even though we identified a recent population bottleneck and expansion based on our summer flounder SFS, additional simulation‐based studies will be useful for more clearly delineating the power to detect demographic fluctuations that have occurred only a few generations in the past with SFS‐based or other methods based on linkage disequilibrium, runs of homozygosity or identity by descent (Gattepaille et al., 2013).

Although our results highlight the promise of genetic data for detecting changes in population size, characteristics of the population of interest or violation of model assumptions can impact the SFS and subsequent inferences. For example, we utilized summer flounder from different sampling locations to increase our sample size in the more recent cohorts in order to increase our power for detecting a recent population size change. However, using fish from different locations may contribute to slight population differentiation, which could artificially increase the number of rare alleles in the SFS and influence downstream demographic inferences (Städler et al., 2009). This effect would appear in the most recent cohorts, though our observations instead suggested a slight deficit of singletons in the 2008 cohort. Summer flounder have also been found to have high rates of dispersal across their species range and no evidence for subpopulations with divergent allele frequencies that could be the source of migrants with different allele frequencies has been found (Hoey & Pinsky, 2018). We also did not detect any intracohort population structure in these data. Still, the possibility of subtle, undetected population structure exists. Similarly, we also tested for but did not find any temporal outliers, yet small increases in allele frequencies due to ecological or evolutionary processes over time could potentially influence our estimates of *N*_e_.

An additional point of consideration is that the fastsimcoal2 program is based off of the Kingman (1982) coalescent, which assumes discrete generations and small reproductive variance under the Wright‐Fisher model (Fisher, 1930; Wright, 1931). However, many species violate these assumptions and alternative models might better reflect biological reality. For example, many marine species, including summer flounder, are characterized by overlapping generations and large variance in reproductive success. Overlapping generations result in slower rates of coalescence, though time rescaling can approximate the Kingman coalescent (Kaj et al., 2001). Overlapping generations in combination with population size changes also lead to an increase in the neutral substitution rate, which could potentially affect the SFS (Balloux & Lehmann, 2012). A recent computationally efficient framework that allows for overlapping generations (Kamm et al., 2017) might hold promise for improving SFS‐based demographic estimates for species that violate assumptions of the Wright‐Fisher model. Violating the assumption of small variance in reproductive success leads to star‐shaped genealogies, and the resulting SFS has an excess of rare and common variants when compared to the expected number under the Kingman coalescent (Eldon & Wakeley, 2006; Tellier & Lemaire, 2014). Under the Kingman coalescent, an excess of rare variants is often interpreted as a population expansion, but this interpretation can become muddled for species with strong sweepstakes reproduction. Multiple merger coalescent models can incorporate variance in offspring number by allowing more than two lineages to coalesce, resulting in a genealogy that is not a binary tree (Tellier & Lemaire, 2014). Even though aspects of summer flounder biology depart from traditional Wright‐Fisher assumptions, our reported estimates of *N*_e_ are probably not strongly biased because many summer flounder individuals do not survive into the next generation.

Additionally, errors in estimating the SFS can influence downstream demographic inferences, though temporal samples can increase the statistical power for detecting past demographic events (Ramakrishnan et al., 2005). We took advantage of archived specimens for improved inferences of historical summer flounder population size changes, but this also introduced differences in sample sizes over time. In particular, we had more limited ability to detect rare alleles in the early cohorts. Though fastsimcoal2 accounts for sample size when simulating the SFS and calculating likelihoods (Excoffier et al., 2013), increasing the number of individuals from the earliest cohort would probably have helped to improve the precision of our inferences (Keinan & Clark, 2012; Robinson et al., 2014). This is a common problem since the availability of archived samples for non‐model organisms is often limited. For RADseq data, bioinformatic choices often reflect a tradeoff between data quality and quantity (Matz, 2018; Shafer et al., 2017). Bioinformatic pipelines employing de novo approaches may result in a high number of singletons and make demographic inference difficult (Shafer et al., 2017). Very rare alleles may also result from sequencing or bioinformatic errors (Johnson & Slatkin, 2008), potentially overrepresenting rare variants in the SFS and influencing demographic conclusions. In addition, aligning RADseq data to a reference genome can lead to more consistent demographic estimates (Shafer et al., 2017), but a genome is not yet available for summer flounder. Null alleles can also bias population genomic statistics and affect the distribution of alleles in the SFS (Arnold et al., 2013; Gautier et al., 2013). Null alleles in RADseq occur when a mutation in a restriction enzyme recognition sequence results in an unrecognized cut site, causing the RAD tag to not be sequenced. This can either result in missing data or heterozygous individuals being falsely identified as homozygotes due to allelic dropout. In order to minimize the number of null alleles without distorting the SFS, Matz (2018) suggests applying a bioinformatics filter requiring that variants be present in a high proportion of individuals, similar to the proportion of missing data filter that we applied. Another study showed that rare variants were common when loci with no missing data were present in the SFS, but that the SFS was characterized by a greater number of variants with intermediate frequencies when loci with missing data due to null alleles were included (Arnold et al., 2013). As a result, Arnold et al. (2013) suggest limiting loci to only those with complete data, but this strategy may inadvertently favour loci that have experienced recent positive selection or strong purifying selection. In addition, Gautier et al. (2013) report that null alleles tend to affect DNA sequences containing ancestral alleles, which are often at high frequency themselves, thus artificially inflating the minor allele frequency and making rare alleles appear more common in the SFS than they actually are. Although null alleles can lead to biases, they can be challenging to identify and attempts to remove them may also have unintended consequences. For estimating summer flounder demography, we removed variants missing in a high proportion of individuals, removed loci with missing data and combined this with tests for temporal outliers to detect loci that may have undergone recent selection. Evidence also suggests that populations with very large effective sizes (*N*_*e*_ > 10^5^) are more likely to be affected by null alleles (Gautier et al., 2013). Even though our SNP data set may have contained null alleles due to the slightly elevated *F*_IS_, particularly for the 1994 cohort, we estimated the 2008 summer flounder effective population size to be between 5859–37,013 individuals, suggesting that null alleles may not be strongly influencing our conclusions in summer flounder.

While it is clear that *N*_e_ recovered after the bottleneck, our analysis was unable to determine if recovery to the prebottleneck effective size was achieved, in spite of census population size achieving the corresponding level of recovery. If present, differences in effective vs. census recovery from historically intense fishing pressure could result from a combination of anthropogenic and biological factors. First, harvest reduces the number of adults contributing to the next generation and results in lower *N*_e_ (Kuparinen et al., 2016; Therkildsen et al., 2019). Even though summer flounder are currently (post‐1990) fished less intensely than in the past, management choices may bias reproductive success in a way that has kept *N*_e_ low. For example, female summer flounder grow faster (King et al., 2001) and mature at a larger size than males (Morse, 1981). Summer flounder harvest is regulated by a minimum length limit, resulting in a higher probability of catching a female at a given age (Morson et al., 2015). The increased fishing mortality for females may have skewed the sex ratio and kept *N*_e_ from recovering as fast as census size. In addition, we used a constant generation length of 2 years over time for demographic modelling, which may influence estimates of bottleneck timing and length. Summer flounder generation length can be challenging to estimate due to serial spawning and a limited understanding of how age influences egg production and hatching success rate. Recovery of the summer flounder fishery was also accompanied by an increased proportion of older, larger individuals in the population (Bell et al., 2014; Terceiro, 2016). These older, larger individuals probably contributed disproportionately to the next generation, leading to a higher generation length as the fishery recovered. Older and larger fish could have also resulted in increased variance in reproductive success among individuals, reducing the ratio of *N*_e_ to census size and preventing *N*_e_ from recovering as much as census size (Barneche et al., 2018; Kuparinen et al., 2016).

Most estimates of *N*_e_ in marine species are one or more orders of magnitude smaller than census size, though particularly small ratios of *N*_e_ to census sizes have been called into question (Hauser & Carvalho, 2008; Hoarau et al., 2005; Waples, 2016; Waples et al., 2018). Using our point estimates of *N*_e_ obtained from demographic modelling with their associated uncertainty and the maximum number of adult breeders (*N*_c_) in the equivalent year, we estimated the *N*_e_/*N*_c_ ratio with the corresponding 95% CIs for summer flounder to be 2.49 × 10^−5^ (4.22 × 10^−6^–5.37 × 10^−5^) and 1.79 × 10^−4^ (1.03 × 10^−4^ 6.49 × 10^−4^) in 1984 (end of the bottleneck) and 2008, respectively. The *N*_e_/*N*_c_ ratio for 1980 (prior to the bottleneck) could not be calculated because the fisheries data set that we used to estimate abundance begins in 1982. Keeping in mind all of the challenges associated with estimating *N*_e_ and *N*_c_, these ratios suggest the convergence of *N*_e_ and *N*_c_ over time, despite the overall increase in census size as the fishery recovered over this timeframe. In general, our estimates of contemporary *N*_e_ were relatively large over time, even after the population declined. These large sizes probably contributed to the temporally stable genetic diversity that we observed among larval cohorts. Although estimates of genetic diversity prior to the bottleneck were unavailable, our estimates during and after the bottleneck suggest that genetic diversity has not increased. In general, genetic diversity can increase in a population through gene flow or through novel mutations. Summer flounder are unlikely to have benefited from gene flow, however, because they are essentially panmictic across their species range (Hoey & Pinsky, 2018). In addition, accumulating novel mutations is a slow process that is unlikely to have had much impact to date (Charlesworth, 2009). Continued monitoring of summer flounder would be useful to understand if effective population size continues to track changes in census size, especially since the population continues to be exploited.

### 4.1 Conclusions

The availability of both demographic data and archived specimens over time is relatively rare and provided an opportunity to compare genetic estimates of demography with known population history in an important fishery species, summer flounder. Temporal samples corresponding to different points in the population history of summer flounder probably aided in the inference of demography over time. Thus, SNP data and coalescent‐based demographic modelling were useful for detecting changes in the magnitude and timing of contemporary population dynamics in summer flounder. Fisheries species are some of the most well‐studied wild populations and include a wide diversity of life history strategies and population histories. When coupled with long‐term collections and molecular methods, these data sets provide valuable opportunities to test genetic and evolutionary theory and illustrate the value of combining existing data sets. For summer flounder in particular, we detected a substantial decline in effective population size followed by growth and recovery of *N*_e_. Genetic methods can provide useful and independent approaches for estimating population dynamics in species of concern, even for large marine populations.

## AUTHOR CONTRIBUTIONS

Jennifer A. Hoey, Malin L. Pinsky, and Kenneth W. Able designed the study; Jennifer A. Hoey organized the samples and prepared the ddRADseq libraries; Jennifer A. Hoey and Malin L. Pinsky designed analysis methods; Jennifer A. Hoey performed the bioinformatics and analyses; all authors discussed results; Jennifer A. Hoey wrote the manuscript; all authors edited the manuscript.

## CONFLICT OF INTEREST

The authors declare no conflict of interest.

## BENEFIT‐SHARING STATEMENT

The benefits generated from this research accrue from the sharing of our data and results on public data sets as described above.

## Supporting information


Appendix S1

## Data Availability

Raw sequencing reads and metadata for summer flounder individuals associated with this project have been archived in the NCBI Sequence Read Archive (SRA) database (BioProject accession No. PRJNA750099). Individual genotypes, other data, and code associated with this study (Hoey et al., 2022) have been made available at GitHub (https://github.com/pinskylab/NePADE) and Zenodo (https://doi.org/10.5281/zenodo.7055607).
