# Supplementary material for: Genetic decline and recovery of a demographically rebuilt fishery species
Source: Mol Ecol. 2022 Oct 11;31(22):5684–98. doi: 10.1111/mec.16697 (PMC9828022; doi:10.1111/mec.16697)
Supplement: Supplementary file 1 — Appendix S1 [file MEC-31-5684-s001.pdf]

MOLECULAR ECOLOGY

Supplemental Information for:

Genetic decline and recovery of a demographically rebuilt fishery species

Jennifer A. Hoey, Kenneth W. Able & Malin L. Pinsky

Table of Contents:

**Table S1: Demographic parameter estimates from *fastsimcoal2* for larval summer flounder using models described in Fig 2.**	Page 1
**Table S2. Relative likelihood of the models described in Fig 2.**	Page 2
**Figure S1. Summer flounder generation length calculated over time.**	Page 3
**Figure S2. Boxplots showing the range of parameter estimates for the best-fit demographic model.**	Page 4
**Figure S3. Confusion matrix resulting from a power analysis that shows the number of times the inferred model corresponded with the known generating model.**	Page 5
**Figure S4. Simulated demographic histories based on the ML parameter estimates for each model in Fig 2 and the inferred history of 10 pseudo-observed SFSs for each model.**	Page 6
**Figure S5. Simulated demographic histories based on the ML parameter estimates of the best-fit model and the inferred history of 50 pseudo-observed SFSs with equal sample sizes across cohorts.**	Page 7
**Figure S6. Minor allele SFSs for the observed dataset and the three best-fit demographic scenarios by larval cohort.**	Page 8
**Figure S7. Expected minor SFS by larval cohort based on the best-fit model.**	Page 9
**Figure S8. Expected minor SFS by larval cohort based on the best-fit model with equal sampling sizes across cohorts.**	Page 10
**References**	Page 11

Table S1. Demographic parameters for larval summer flounder estimated in *fastsimcoal* v.2.6 using models described in Figure 2. These analyses utilized the observed dataset of 1,068 loci across 279 summer flounder larvae. Population size estimates have been converted to diploid units and time estimates are in generation time (calculated to be 2 years for summer flounder). Parameter estimates were obtained from the *fastsimcoal* run with the maximum likelihood.

**Model**	**NANC**	**TCAR**	**TLEN EARLY**	**NBOT EARLY**	**TBOT EARLY**	**NPREBOT**	**TLEN**	**NBOT**	**TBOT**	**NPOP08**
1										10605
2						24670	3	1561	7	2997
3						7250	3	202	12	16530
4	86					60466	2	4314	7	5364
5	25359		1	2680	4	15610	1	690	8	2651
6	1052					32209	2	910	12	10212
7	342	10								63790

Table S2. Relative likelihood, the number of estimated parameters, Akaike Information Criteria (AIC), the difference between model AIC and that of the model with the minimum AIC (ΔAIC), and the relative AIC weight (w) for each of the models described in Figure 2 for summer flounder. These analyses utilized the observed dataset of 1,068 loci across 279 summer flounder larvae.

**Model**	**Maximum ln Likelihood**	**Number of Parameters**	**AIC**	**ΔAIC**	**AIC weight (w)**
1	-7335.020	1	14672.04	482	$2.681167 \times 10^{-105}$
2	-7317.331	5	14644.66	455	2.363360×10^{-99}
3	-7313.416	5	14636.83	447	1.185322×10^{-97}
4	-7095.396	6	14202.79	13	2.111544×10^{-03}
5	-7319.860	9	14657.72	468	$3.452663 \times 10^{-102}$
6	-7089.237	6	14190.47	0	0.9978885
7	-7103.143	3	14212.29	22	1.831797×10^{-05}

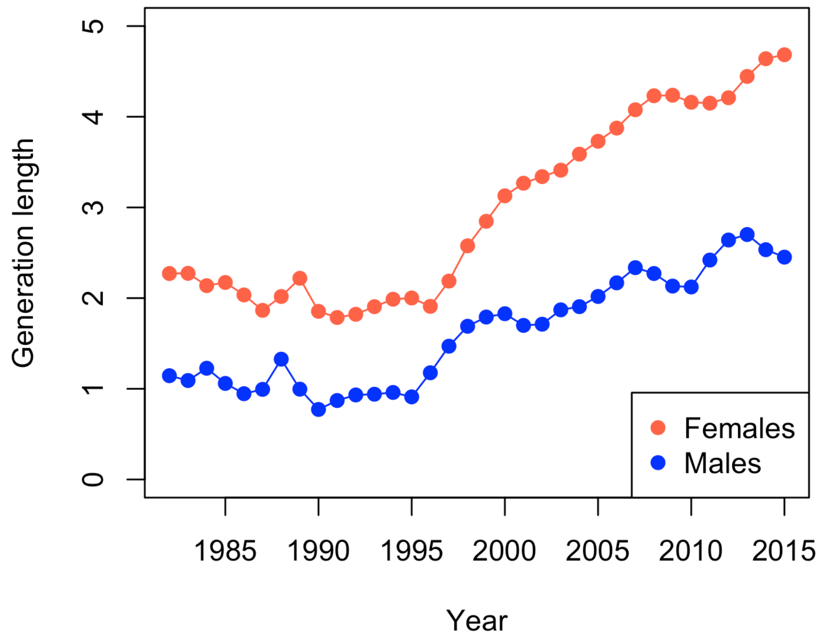

Figure S1. Summer flounder generation length calculated using the abundance and the proprtion of mature fish in each age class from Terceiro (2016), the age-length relationship from Penttila et al. (1989) and length-fecundity curves from Morse (1981).

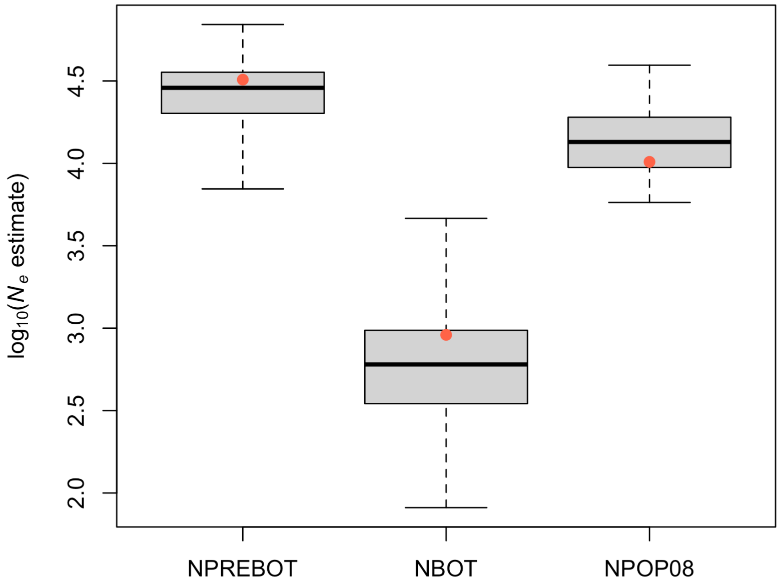

Figure S2. Boxplots showing the median, interquartile ranges and overall ranges of N_e estimates for 100 non-parametric bootstraps for summer flounder. Maximum-likelihood point estimates under the best-fitting model (Model 6) from *fastsimcoal* v.2.6 are shown as red dots. N_e estimates have been converted into diploid units. NPREBOT refers to ancestral N_e prior to the bottleneck; NBOT to N_e during the bottleneck; and NPOP08 to N_e at the time of sampling in 2008.

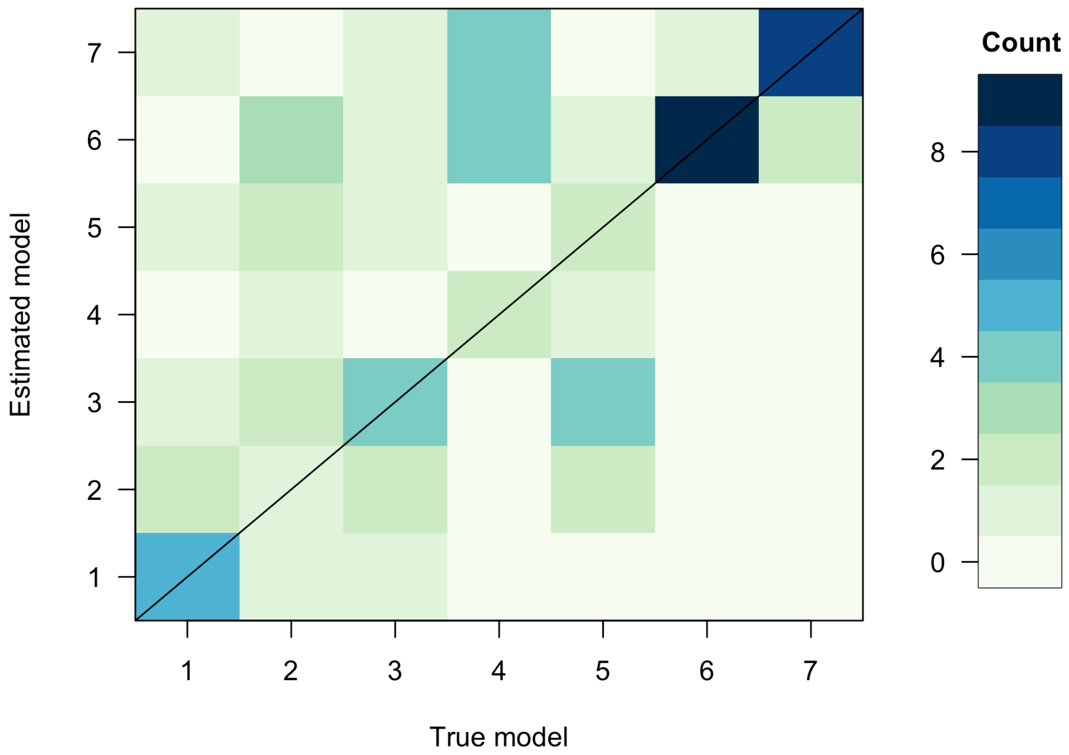

Figure S3. Confusion matrix showing high power for correctly selecting Model 6 if Model 6 is the true model. When the true demographic scenario was Model 4, it was difficult to accurately differentiate between Models 4 and 6, but these models were quite similar. Model 7 was similar to Models 4 and 6 but without a bottleneck, and Model 7 was only rarely confused with Models 4 and 6. These results suggest that, if Model 6 is inferred from an empirical dataset, then summer flounder 1) likely experienced exponential growth of the ancestral population, and 2) this growth was most likely followed by a bottleneck and then an additional increase in population size. For model descriptions, see Figure 2.

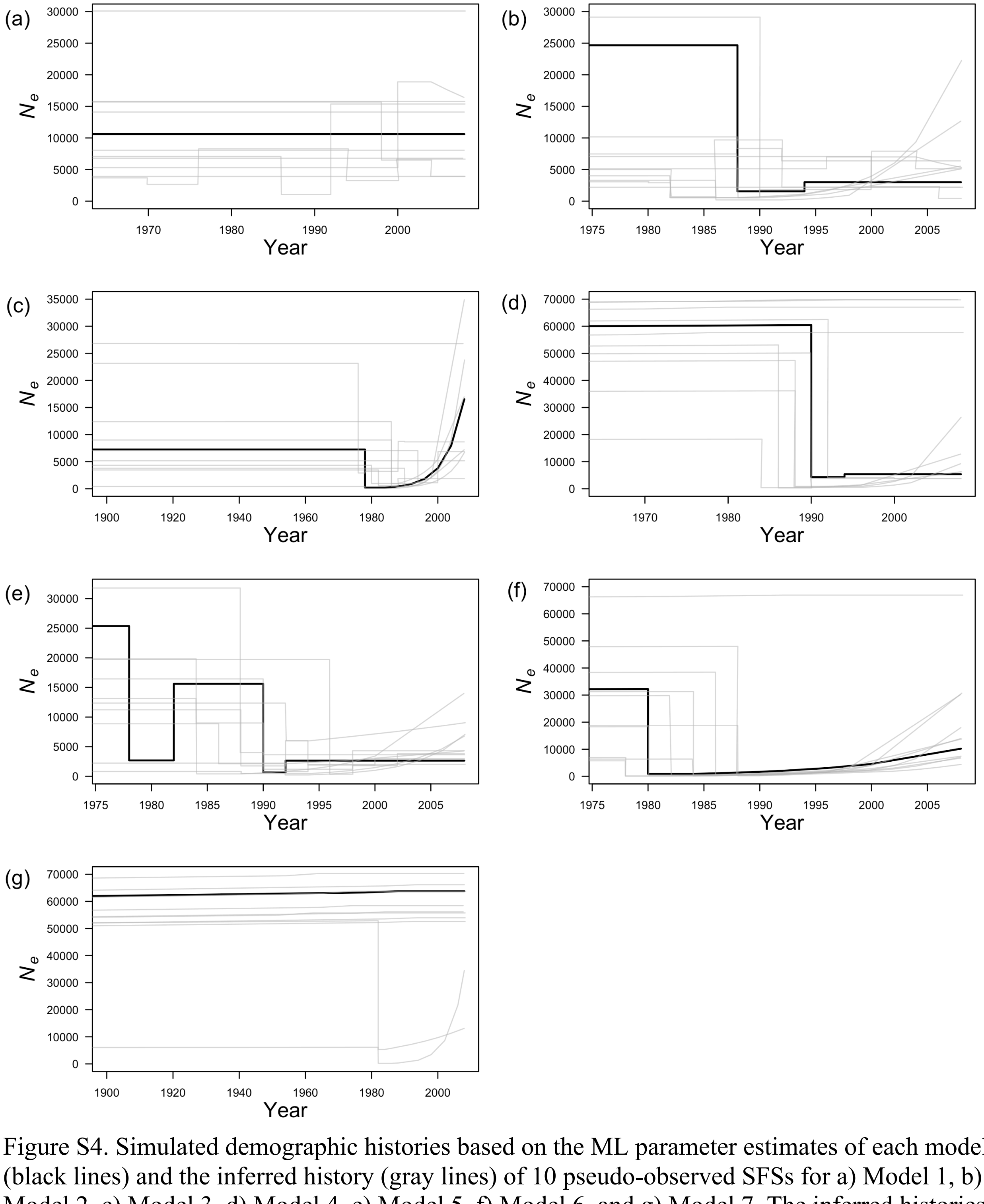

Figure S4. Simulated demographic histories based on the ML parameter estimates of each model (black lines) and the inferred history (gray lines) of 10 pseudo-observed SFSs for a) Model 1, b) Model 2, c) Model 3, d) Model 4, e) Model 5, f) Model 6, and g) Model 7. The inferred histories suggest that recent demographic events can be inferred using SFS-based methods. For model descriptions, see Figure 2.

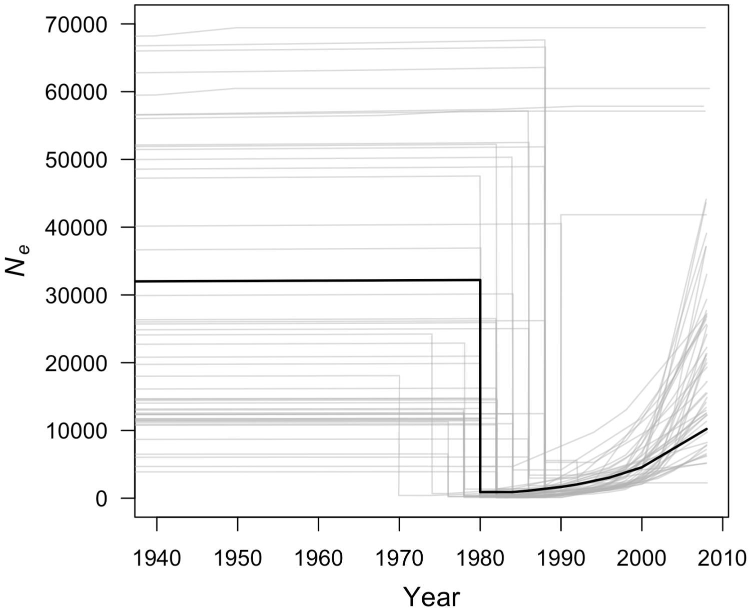

Figure S5. Simulated demographic histories based on the ML parameter estimates of Model 6 (black line) and the inferred history (gray lines) of 50 pseudo-observed SFSs where 80 individuals were sampled per cohort. Across the 50 simulations, 44 (88%) correctly inferred a bottleneck and recovery represented by Model 6. The inferred histories suggest that we are able to recover the true demographic history when it is known, even when sample sizes across cohorts are equal and differences in the SFS among cohorts are small. For model descriptions, see Figure 2.

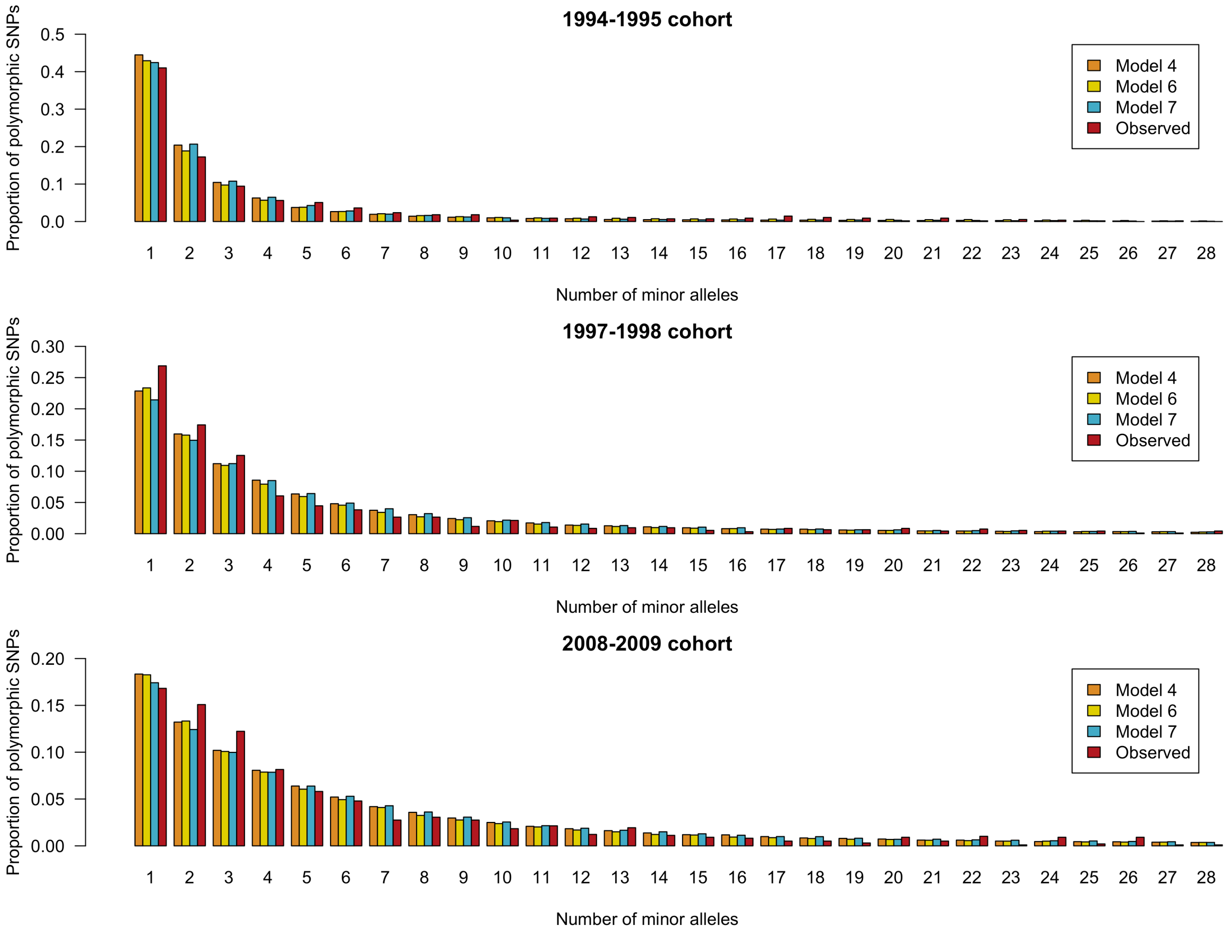

Figure S6. Minor allele site frequency spectra (SFS) for each larval summer flounder cohort comparing the distribution of minor alleles under the three best-fit demographic scenarios to that of the observed using a dataset composed of 1,068 loci across 279 larvae (26 larvae in the 1994-1995 cohort; 103 in the 1997-1998 cohort; and 150 in the 2008-2009 cohort). The SFSs for the 1997 and 2008 larval cohorts have been truncated for easier visualization. See Figure 2 for full descriptions of tested demographic scenarios.

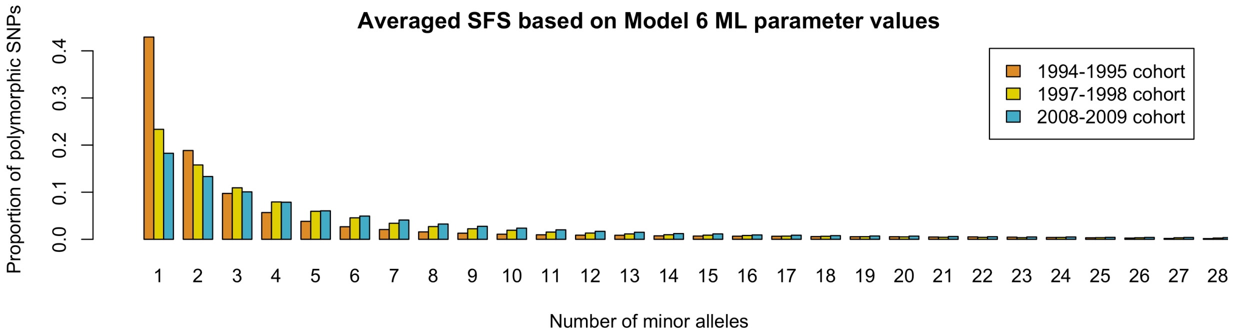

Figure S7. Expected site frequency spectra (SFS) of each larval cohort comparing the distribution of minor allele frequencies averaged across 100 *fastsimcoal2* simulations using the ML parameter values for Model 6 reported in Tables 2 and S1. The mean and standard deviation of the number of polymorphic loci were 570.8 ± 17.2, 899.9 ± 12.1 and 962.3 ± 10.1 for the 1994-1995, 1997-1998 and 2008-2009 larval cohorts, respectively. The SFSs for the 1997 and 2008 larval cohorts have been truncated for easier visualization.

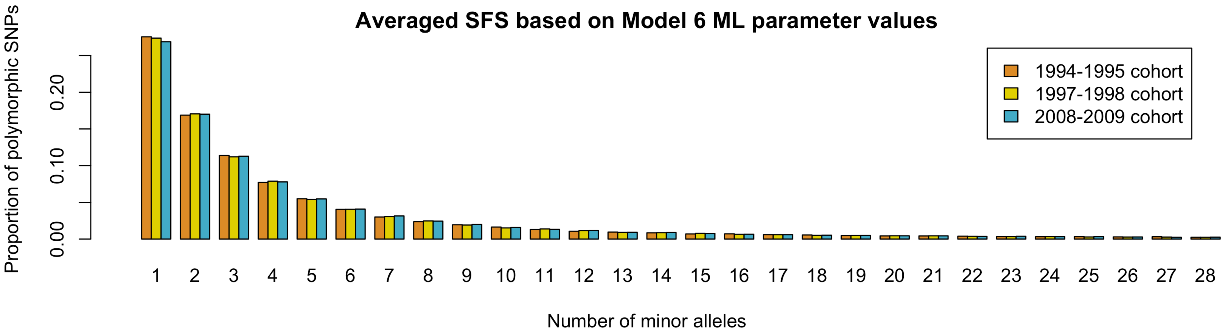

Figure S8. Expected site frequency spectra (SFS) of each larval cohort based on Model 6 ML parameter values with 80 diploid individuals sampled in each cohort at 1068 loci. Each SFS represents the distribution of minor alleles averaged across 100 *fastsimcoal2* simulations. The ML parameter values for Model 6 used to generate these SFSs are reported in Tables 2 and S1. While differences in the SFS among larval cohorts is less apparent than with unequal sampling (see Figure S7), important differences remain. When combined with simulation testing (Figures S3, S4, and S5), these results demonstrate that a serial sampling scheme results in strong inferential power for determining demographic history, regardless if equal (see Figure S5) or unequal numbers of fish are sampled in each cohort (see Figure S4f for comparison).

References

Morse, W. W. (1981). Reproduction of the summer flounder, *Paralichthys dentatus* (L.). *Journal of Fish Biology*, *19*(2), 189–203. https://doi.org/10.1111/j.1095-8649.1981.tb05823.x

Penttila, J. A., Nelson, G. A., & Burnett, J. M. (1989). *Guidelines for Estimating Lengths at Age for 18 Northwest Atlantic Finfish and Shellfish Species*. 45.

Terceiro, M. (2016). *Stock Assessment of Summer Flounder for 2016*.
